# A decade of independent prescribing in the UK: a review of progress

**DOI:** 10.1186/s13047-022-00541-8

**Published:** 2022-05-11

**Authors:** Matthew TJ Fitzpatrick, Alan M Borthwick

**Affiliations:** 1 School of Clinical Sciences, QUT, Brisbane, Australia; 2 School of Health Sciences, Faculty of Environmental and Life Sciences, University of Southampton, Southampton, UK; 3 School of Health and Human Sciences, Southern Cross University, Lismore, Australia

**Keywords:** Independent prescribing, Statutory exemptions, Controlled drugs, Commission on Human Medicines, Advisory Council on the misuse of drugs, Misuse of drugs regulations, Human Medicines Regulations, Medicines Act, Misuse of Drugs Act

## Abstract

**Background:**

2022 marks a decade since the profession of podiatry secured independent prescribing rights in the UK. Widely viewed as a significant milestone, its advent appeared to herald a new age of practice, with increased autonomy, a broader scope of practice and improved patient care. Access to any medicine within the British National Formulary (with a few notable exceptions) seemed to signal an end to the perennial difficulties and frequent disappointments in obtaining ease of access to medicines necessary for effective practice.

**Main body:**

Recent attempts to expand the scope of prescribing practice to include access to a broader range of controlled drugs (CDs) have led to unanticipated complications which may even threaten existing rights. These issues highlight the limitations of current independent prescribing and the continuing inability of podiatrists to access certain key medicines, primarily controlled drugs. Reliance on specified ‘lists’ of approved medicines, whether a controlled drug list for prescribers or the use of statutory exemptions by non-prescriber podiatrists, remain inflexible and difficult to change. The data underpinning much of this paper is derived from the work undertaken by the authors as representatives of podiatry on NHS England’s Chief Professions’ Officers’ Medicines project, in particular involving submissions to the Commission on Human Medicines and the Advisory Council on the Misuse of Medicines, spanning the years 2017–2021. It describes a complex process, and highlights a misalignment between two legislative frameworks that threaten to unravel existing rights.

**Short conclusion:**

Ongoing difficulties relating to controlled drugs illustrate the problematic nature of current supply, administration and prescribing rights in podiatry. Efforts to keep pace with periodic legal reclassifications of medicines are constrained by limited and inflexible legal mechanisms, and formal approval for extended access via prescribing remains unpredictable and complex. For prescriber and non-prescriber^1^ (^1^Non- prescriber podiatrists are those who are neither supplementary or independent prescribers, but do enjoy existing administration and supply rights to certain medicines.) podiatrists alike, the profession of podiatry faces a new challenge to its ability to access medicines, and to realise its full clinical potential.

## Background and context

The Medicines Act (1968) enshrined a series of changes which afforded greater public safeguards in the governance of medicines, whilst also imposing wider restrictions on those who might legitimately supply, prescribe or administer them [1]. Introduced in response to the thalidomide scandal, the Act reflected a clear need to strengthen governance through legislation [2, 3]. The recommendations of the Dunlop Committee of 1962 ultimately led to new legislation which brought together an array of separate laws under one umbrella, in the form of the Medicines Act (1968) [3]. One of its unintended consequences was the exclusion of a number of healthcare professions from the provisions enabling access to the new categories of medicines outlined within the Act 1. As a consequence, access to medicines was tightly controlled and ultimately constrained. Podiatric access would require the professional body to formulate a clear rationale to persuade the existing establishment of the safety, competence, ability and governance of its procedures, as well as presenting a firm case of clinical need in support of its claims [1]. Thus, at the outset, the non-medical /non-dental professions were disadvantaged, and had to work towards acceptance and recognition over many years [4].

Convincing the key regulatory and governing authorities – the Medicines Commission and its successors - proved a long and arduous task. Whilst small concessions were periodically granted over time, in the form of approved lists of a handful of medicines, the mechanisms involved fell far short of the right to prescribe medicines and allowed only limited rights to administer or supply each medicine [1, 4, 5]. Furthermore, these mechanisms were unable to keep pace with changes to the reclassifications of medicines, or the advent of newer medicines [6]. Statutory Instruments (SIs) providing ‘exemptions’ (that is, exemptions from restrictive legislation) were used to enable access to some ‘pharmacy only’ and ‘prescription only’ medicines by podiatrists. Statutory Instruments are legislative tools known as secondary or delegated legislation, which allow for minor amendments to the main Act (The Medicines Act 1968), approved by a government Minister [7]. Two types were used; those that permitted rights to supply a given list of medicines 2, and those that allowed rights to administer certain specified medicines^3^. Over the years, several such instruments were laid, and on each occasion were preceded by a complex and arduous process lasting over a year before a small number of additional medicines were added to the lists [4, 8–10]. They foreshadowed the complex processes involved in attaining independent prescribing [11].

Only in 1999 was the prospect of actual prescribing formally proposed for certain allied health professions, as a solution to the emerging workforce and demographic challenges facing the National Health Service [12, 13]. The Crown Report suggested 5 key non-medical professions as early candidates for independent prescribing, including podiatry [12]. In 2005 suitably qualified podiatrists became ‘supplementary’ prescribers, affording a limited autonomy in prescribing practices 4. But it was not until the summer of 2013 that podiatry, alongside physiotherapy, became the first of the allied health professions to be granted legislative rights as independent prescribers [6, 11] 5. Although in no sense equivalent to medical prescribing, it offered a considerable advance in clinical autonomy and appeared to signal a huge step forward in the ability of podiatrists to fully manage patients.

In the decade since the introduction of independent prescribing, much of the literature has focused on the uptake of prescribing by AHP practitioners within each profession, alongside the evaluation and impact of its initial implementation [14–20]. Some papers have examined specific obstacles to independent prescribing within allied health [21, 22], whilst others have focused on specific medicines used or conditions treated [23–25]. However, little attention has been paid to the inherent limitations of the legislative frameworks, or their impact on the effectiveness of prescribing, with a very few notable exceptions [26–30]. Of these, three in particular merit further mention, given their relevance to the current case of podiatry [28–30]. Graham-Clarke et al. [26] focused on White and Green papers and various related regulatory and professional body documents, and provided a glimpse of the complex processes involved, and Raghunandan et al. [27] outlined the new legal categories of prescriber in New Zealand. However, it is Gallagher’s papers that drill down to the core problem in terms of the legal frameworks and their implications – particularly their inadequacies [28–30]. Clear obstacles to the effectiveness of current independent prescribing practices in allied health have exposed legislative contradictions [28–30], and changes to the classification of medicines have affected existing supply and administration lists for non-prescribing podiatrists [25]. This paper seeks to identify the key challenges to the access of medicines facing the profession of podiatry, and their basis in legislation. It brings to light the shortcomings of the legal frameworks, and the need to rely on repeated approval processes to maintain the status quo, let alone enhance existing practice.

## Main text

### Independent prescribing and contemporary access to medicines

Far from enabling the delivery of seamless care, independent prescribing appears to maintain limits to practice that constrain the scope of the profession [29, 30]. This is manifest in two exemplars which have necessitated further submissions to the Commission on Human Medicines (an advisory, non-departmental body sponsored by the Department of Health and Social Care) and the Advisory Council on the Misuse of Drugs (sponsored by the Home Office) over the last year (2021). Both concern ‘controlled drugs’, a specific category of ‘prescription only medicines’ that is governed not only by the terms of the Medicines Act and its statutory amendments (The Human Medicines Regulations 2012), but also by the Misuse of Drugs Regulations (2001) and its underpinning primary legislation, the Misuse of Drugs Act (1971) 6.

Controlled Drugs are drugs that are subject to “high levels of regulation as a result of government decisions on drugs that are considered to be especially addictive and harmful if misused” 7. They are subject to “additional legal controls as they carry a higher risk of being misused” 8. Within the terms of the Medicines Act and Human Medicines Regulations, they are classified as ‘prescription only medicines’, but are given special status within the Misuse of Drugs Regulations as ‘controlled drugs’. The Misuse of Drugs Act (1971) categorises drugs in classes (A, B and C), graded broadly according to the harmfulness attributable to a drug when it is misused. In the Misuse of Drugs Regulations, however, schedules are used to define who may supply and possess categories of medicines (schedules 1 to 5) where schedule 1 drugs pose the greatest risks, and schedule 5 the least risk. The list for podiatrists largely addresses medicines in classes B and C, and schedules 3 to 5 (as indicated in the Statutory Instruments).

Whilst changes to the Medicines Act require the approval of the Commission on Human Medicines (CHM), changes in access to controlled drugs also require the approval of the Advisory Council on the Misuse of Drugs (ACMD). In short, access to controlled drugs requires dual approval, from both the Department of Health and Social Care, and the Home Office.

To provide further context, the work of Gallagher merits mention. Gallagher highlights incompatibilities between the medicines and the misuse of drugs regulations affecting therapeutic radiographers and paramedics, as well as physiotherapists and podiatrists [28–30]. His work identifies discrepancies that help, in part, to account for the issue facing podiatry. He contends that the apparently irreconcilable differences between the Human Medicines Regulations (2012) (HMRs) and Misuse of Drugs Regulations (2001) (MDRs) relating to the allied health professions actually impose restrictions where none are needed. This is premised on the assertion that two amendments to the MDRs were “added in error” (notably regulations 6B and 6 C), and that the subsequent professional and regulatory bodies guidance remains a “misrepresentation of the law as written”. Such a serious assertion demands attention, and on closer inspection accounts in part for the problems which have recently beset podiatry. The disjunction between the MDRs and the HMRs affected the paramedic profession in 2018, as the HMRs were amended to allow paramedic independent prescribing, but, crucially, no corresponding amendment was made to the MDRs [29, 30]. However, the key to the problem, for Gallagher, is the fact that the Misuse of Drugs Act (1971) “prohibits possession, production, supply, import and export of CDs…as allowed by regulations”, but, crucially, does “not prohibit the act of prescribing CDs” [29, 30]. No restriction was placed on doctors or dentists, yet the advent of nurse and pharmacy independent prescribing saw the introduction of a new amendment to the MDRs – regulation 6B – which gave “authority” for these groups to prescribe CDs, when it was not actually required. Subsequently, when physiotherapy and podiatry were granted independent prescribing, another “authority” was granted in the MDRs – regulation 6 C – which is also deemed unnecessary [29, 30]. For podiatry, however, there were even further implications, which were not revealed in Gallagher’s work, but which came to light in late 2019, uncovered by the authors of this paper. These relate to the mistaken “revocation” of an existing “Group Authority” 9 for podiatrists with supply rights annotated as POM-S by the regulatory body, the Health and Care Professions Council (HCPC), to supply co-dydramol 10. In addition, whilst the HMRs were amended (2011) to add codeine phosphate and co-codamol to the lists, no corresponding amendment was made to the MDRs.

Each of the two exemplars presented below highlight separate problems which undermine the ability of the allied health professions to fully utilise their skills within the current legislative frameworks available. The first concerns the need to respond to changes in the classification of medicines within the terms of the MDRs, and the second relates to a disjuncture between the medicines legislation approved by the CHM and the Misuse of Drugs Regulations (and amendments) by the ACMD. The former outlines the ongoing difficulties in persuading the authorities that the profession requires additional access as part of the broadening scope of modern practice, the latter highlights the critical impact on practice of a misalignment between the two separate sets of legislative requirements. In combination they constitute a significant obstacle to maintaining existing practice, as well as potentially preventing further developments in clinical practice.

#### 1. Reclassification of medicines: the unintended consequences for practice

In line with the establishment of independent podiatrist prescribing in 2012, access to a limited list of controlled drugs (CDs) was approved by the ACMD (Home Office). It enabled independent prescriber podiatrists access to 4 CDs (diazepam, lorazepam, dihydrocodeine and temazepam). At the time, tramadol (an ‘alternative analgesic approach’ in the management of pain ^10^) was commonly prescribed by independent prescriber podiatric surgeons as part of post-surgical pain management. However, in 2014 it was reclassified as a schedule 3 controlled drug. As a result, independent prescriber podiatrists could no longer prescribe it, as it was not one of the four approved drugs specified in the list.

By reclassifying tramadol as a controlled drug, the ACMD unwittingly yet effectively deprived podiatric surgeons of their ability to fully manage post-surgical cases 11. Alternative measures were required, which in practice meant relying on physicians to prescribe the drug, or resorting to supplementary prescribing (requiring physician approval), or even to contemplate prescribing more potent medication earlier than would otherwise have been indicated. This was not in keeping with established evidence-based practice, thus ensuring the change in classification would present both a clinical and possibly ethical dilemma for the clinician.

In response, the professional body (Royal College of Podiatry) was forced to seek a remedy, requiring a further submission to the CHM and ACMD in order to re-establish the status quo (and reinstate access to tramadol). Three further controlled drugs were added, as a result of yet further changes, notably the reclassification of gabapentin and pregabalin (both used by prescriber podiatrists) as CDs in 2019. A new submission was prepared and finally submitted to the Commission on Human Medicines in July 2021 by the Chief Professions’ Officers’ medicines team, on behalf of the profession.

#### 2. Codeine phosphate, co-codamol and co-dydramol: further unintended consequences for practice

Over the same timeframe, starting in late 2019, the Medicines and Medical Devices Committee of the Royal College of Podiatry identified a problem requiring the intervention of the Home Office, ultimately leading to another submission before the ACMD. It transpired that certain changes to the regulations had taken place, unbeknown to the Royal College of Podiatry, which held considerable implications for podiatry practice across the nation. These changes comprised a ‘revocation’ of an existing access right, and a failure to include within the MDR regulations further rights which had been approved by the Commission on Human Medicines for inclusion in the Human Medicines Regulations. Whilst the Human Medicines Regulations (2012) recognised supply rights (for podiatrists with the HCPC ‘POM-S’ annotation) to codeine phosphate, co-codamol and co-dydramol, approval from the ACMD had been withdrawn in the case of co-dydramol, and omitted from the MDRs altogether in the case of codeine phosphate and co-codamol. In short, podiatrists across the country were supplying these products to their patients without realising that approvals outlined in the Human Medicines Regulations had been revoked or omitted from the Misuse of Drugs Regulations. Advice from the Home Office to the authors suggested that the latter trumped the former (a point contested by Gallagher [30]).

Following amendment of the MDRs in 2015 (which recognised independent prescriber podiatrists access to four controlled drugs), measures were put in place to revoke an existing Group Authority for co-dydramol, on the assumption that its use would be superceded by the new list of 4 CDs available to independent prescriber podiatrists ^9^. Equally, it then appeared that codeine phosphate and co-codamol, which had been approved by the CHM for addition to the access and supply list in the HMRs, were not in fact co-approved by the ACMD and added to the MDRs. This illustrates the vulnerability of a system which appears to require two separate bodies of approval covering two separate sets of legislation. This suggests that better feedback between the two systems is necessary to avert similar obstacles in future. This predicament left the Royal College of Podiatry, as the leading professional body, in a difficult position, in terms of addressing the implications for practice and in ensuring compliance with the regulations.

The fact remains that the misalignment of the HMRs and the MDRs has in reality resulted in a reversal in the ability of podiatrists to prescribe CDs effectively, and may threaten their right to supply schedule 5 CDs.

## Conclusions

Ten years on, independent prescribing has not, after all, proven to be the complete solution to the perennial problems of access to medicines. It is clear that effective solutions are required, and there may be a number of potential options. Each has strengths, limitations, and likely consequences, which merit consideration. One potential option is to amend existing legislation to grant podiatrist (and other AHP) independent prescribers access to all controlled drugs, without requiring a specific and limited list. This would remove the obstacle posed by the reclassification of medicines to controlled drug status, and allow new medicines to be easily accessed. However, present concerns over “opiophobia” [31], and legal cases of misuse in the USA 12 may mitigate against any such ease of access. Current reliance on fixed lists of medicines, whether for supply and administration rights (as in the case of codeine phosphate, for example), or prescribing rights (for any schedule 3 or 4 CDs, for example) suffer from these drawbacks. However, if prescriber AHPs were to be granted open access to controlled drugs, the professions might concede the need to retain non-prescriber access via administration and supply mechanisms, thus streamlining the process and ensuring all such medicines were accessed only by prescribers. Presently, each list may only be adjusted following the preparation and submission of a case for change, duly considered by the Commission on Human Medicines and the Advisory Council on the Misuse of Medicines, a lengthy and often tortuous business.

There is a clear second potential option. Currently, the misalignment of the two differing legislative regulations (MDRs and HMRs) might be addressed either by establishing a precedence between the two, or, better still, by combining the processes under which decisions are made so that one authority presides over each case. This might take the form of a combined ACMD and CHM approval meeting, whenever controlled drugs are included in a submission. This would need to be underpinned by a joint, integrated, set of processes and procedures between the Department of Health and Social Care and the Home Office. It would have the advantage of streamlining the entire process, and ensuring effective communication, but it may be difficult to implement easily. However, as two ‘competing’ frameworks, it is clear that the HMRs and MDRs do not dovetail as they perhaps should, and a joint approval body would presumably go some way to alleviate these issues identified in this paper.

A third option would be to address the matter of the utility of the ‘prescribing authority’ embedded in the MDRs for the non-medical/non-dental professions – regulations 6b and 6c. This would have to involve a clarification of the need for any such authority. If these clauses were indeed “added in error” [30], and unnecessary, then their removal would acknowledge the freedom of AHPs to prescribe CDs without the need for specified lists.

Alternatively, the final option for change would be to undertake an overhaul of the underpinning primary legislation separating the HMRs from the MDRs, although this would require very considerable change, investment in time and resource, and a clear eye on the detail, in order to prevent any further inadvertent obstacles arising. This would clearly be the least likely option, given the demands it would place on the civil service, and the costs that it would incur.

Any option appraisal should sensibly consider the status quo. In this case a ‘no action’ agenda would merely perpetuate the current problems, duplicating effort with the further potential for disagreement over approvals between the two current authorities (CHM and ACMD), with no clarity over which, if any, has precedence. Equally, retaining both prescribing plus the supply and administration annotations, offers both advantages and disadvantages. The former would be served by retaining more than one option for accessing a medicine. The disadvantages are evident in the potential for confusion and in the limitations of the non-prescribing annotations. In short, this ‘option’ is itself a problem rather than a solution, although it is the easiest to adopt as it requires no change. However, as this paper makes clear, ‘no change’ is not a real option, as it is a hindrance to effective practice.

As things stand, it is the fate of the professional bodies to continually plan for submissions that capture the needs of practitioners in a contemporary context, in a bid to keep pace with the development of new medicines and the reclassification of others. In many respects, for the allied health professions it remains a Sisyphean task; and the burden must be borne by the professional bodies.

## Data Availability

Not Applicable (as a review/policy paper).

## Abbreviations

CHM: Commission on Human Medicines
ACMD: Advisory Council on the Misuse of Drugs
HMRs: The Human Medicines Regulations (2012)
MDRs: Misuse of Drugs Regulations (2001)
HCPC: Health and Care Professions Council
POM-S: Prescription Only Medicines (Supply) annotation regulated by the Health and Care Professions Council
CDs: Controlled Drugs
GA: Group Authority (issued by the Home Office)
SI: Statutory Instrument (secondary or delegated legislation)

## Footnotes

1 The three categories comprised: General Sales List (GSL) medicines, Pharmacy Only Medicines (P) and Prescription Only Medicines (POMs), reflecting the relative risks and necessary safeguards for each medicine.

2 The first supply list was included in The Prescription Only Medicines (Human Use) Amendment Order 1998, SI No 108. This was followed by The Medicines for Human Use (Administration and Sale or Supply) (Miscellaneous Amendments) Order 2006, SI No. 2807 and then The Medicines (Miscellaneous Amendments) Order 2011, SI No. 1327.

3 The Medicines (Prescription Only) Order 1980, SI No.1921.

4 The Medicines (Sale or Supply) (Miscellaneous Provisions) Amendment Regulations 2005, SI No. 764.

5 The Human Medicines (Amendment) Regulations 2013, SI No. 1855.

6 In particular, The Misuse of Drugs (Amendment) (No. 2) (England, Wales and Scotland) Regulations 2015, SI No. 891; The Misuse of Drugs and Misuse of Drugs (Safe Custody) (Amendment) (England, Wales and Scotland) Regulations 2014, SI No. 1275; The Misuse of Drugs and Misuse of Drugs (Safe Custody) (Amendment) (England and Wales and Scotland) Regulations 2018, SI No. 1383; Human Medicines Regulations 2012, SI No. 1916, schedule 17, Part 1, p. 236.

7 NHS England: https://www.england.nhs.uk/south/info-professional/safe-use-of-controlled-drugs/.

8 HCPC: https://www.hcpc-uk.org/standards/meeting-our-standards/scope-of-practice/medicines-and-prescribing-rights/controlled-drugs/.

9 A Group Authority is defined thus: “In a handful of specialist circumstances a licence granting a ‘Group Authority’ to a specific organisation or professional body of individuals may be issued. These have been issued in very small numbers in exceptional cases where individually licensing people to handle controlled drugs ‘in the field’ is impractical and disproportionate. The process of considering whether to issue a group authority is complex and taken in conjunction with all relevant parties. Consideration is given on a case-by-case basis and there is no premise in favour of granting a Group Authority.” Home Office Drug Licensing and Compliance Unit Drug Domestic Licensing, Charging and Processing Guidance. 2012.

10 Enquiries by the authors to the Home Office revealed an email dated 8th April 2016, from the Care Quality Commission Medicines Optimisation Team to various appropriate authorities, stating “With effect from 6 April 2016 the Home Office Group Authority issued to (state registered) Chiropodists in October 1992 has been formally revoked. This is in response to changes to the Misuse of Drugs Regulations 2001 during 2015 which allowed limited use of some controlled drug containing medicines to HCPC registered chiropodists and in effect nullified the GA…”.

11 A letter from the Dean of the Faculty of Podiatric Surgery, of the Royal College of Podiatry, was sent to the Medicines and Medical Devices Committee to alert it to the problem, and to seek advice on redressing it (3rd August 2016).

12 The United State Attorney’s Office, Northern District of Georgia, Jury finds podiatrist guilty of operating pill mill, May 9th 2019. URL: https://www.justice.gov/usao-ndga/pr/jury-finds-podiatrist-guilty-operating-pill-mill#:~:text=Arnita%20Avery%2DKelly%2 C%20a%20licensed,Springs%2 C%20and%20Lithonia%2 C%20Georgia. Kelman, B, Nashville podiatrist who gave opioids to ‘anyone,‘ to plead guilty in drug dealing case, The Tennessean, 15th February 2020. URL: https://eu.tennessean.com/story/news/health/2020/02/15/nashville-podiatrist-timothy-abbot-opioid-prescriptions-to-plead-guilty-drug-dealing/4754499002/.
